# Management of a severe abdominal compartment complicating fulminant cardiogenic-septic shock: An abdominal arterio-venous single-tube ECMO bypass saved a young patient’s life after OHCA

**DOI:** 10.1177/02676591221087545

**Published:** 2022-04-10

**Authors:** Julian Kreutz, Amar Mardini, Ann-Christin Schäfer, Bernhard Schieffer, Birgit Markus

**Affiliations:** Department of Cardiology, Angiology and Intensive Care Medicine, University Hospital (UKGM) Marburg, Marburg, Germany

**Keywords:** out of hospital cardiac arrest, intra-abdominal compartment syndrome, management of complications

## Abstract

**Introduction:**

In severe cardiogenic shock, for example, following cardiac arrest, the implantation of an extracorporeal hemodynamic assist device often seems to be the last option to save a patient’s life. However, even though our guidelines provide a class-IIa-recommendation to implant a veno-arterial extracorporeal membrane oxygenation (vaECMO) device in these patients, the accompanying disease- and device-associated complications and their consequences remain challenging to handle.

**Case presentation:**

A 43-year-old patient presented with severe cardiogenic-septic shock with a complicating abdominal compartment due to a prolonged out-of-hospital cardiac arrest (OHCA). A loss of function of the vaECMO, implanted immediately after admission, impended due to increasing intra-abdominal pressure. This dangerous situation was resolved by crafting an experimental “arterio-venous shunt,” using the side port of the reinfusion (arterial) vaECMO cannula and a downstream large-volume central access in the right femoral vein toward the abdominal venous system, which led to the patient’s full recovery.

**Conclusion:**

In patients with cardiogenic shock, the use of catecholamines and implantation of extracorporeal assist devices alone do not ensure successful therapy. To optimize the outcome, device- and disease-associated complications must also be managed in a timely and minimally invasive procedure.

## Introduction

Cardiogenic shock remains one of the leading causes of death.^1^ The 30-day mortality and long-term survival of a patient experiencing cardiogenic shock essentially depend on the underlying cause of their heart failure, their constitution and age, and the accompanying medical complications.^2^ In this highly time-critical situation, the use of extracorporeal life support systems (ECLS) for hemodynamic stabilization as part of the management of cardiogenic shock and out-of-hospital cardiac arrest (OHCA) patients is established particularly in tertiary centers worldwide. The aim is to prevent the “vicious circle” of cardiogenic shock resulting in heart and organ failure. However, the increased use of extracorporeal life support systems (ECLS) also creates new challenges for medical professionals in managing both shock- and device-associated complications. In this situation, rapid and precise management is crucial.

The current paper presents the case of a 43-year-old patient suffering from OHCA due to fulminant myocarditis. High-dose catecholamine therapy was administered for at least 12 h before admission to the intensive care unit (ICU). After implantation of vaECMO in cardiogenic shock, a complicating abdominal compartment led to a fulminant loss of function of venous ECMO backflow with impending circulatory failure and death of the patient. In this situation, the maximal thoracic position of the drainage (venous) ECMO cannula inflow and the experimental implementation of a retrograde arterio-venous bypass toward the abdominal venous system via the reinfusion ECMO side-port and the V. femoralis led to a resumption of vaECMO function, hemodynamic stabilization, and, in the end, survival of this critically ill, but very young patient.

## Case presentation

We present the case of a 43-year-old patient suffering from prolonged cardiogenic-septic shock. In December 2019, he was transferred from a smaller district hospital to the University Hospital in Marburg and admitted to the intensive care unit (ICU).

In brief, the patient had collapsed at his workplace a day before. A bystander cardio-pulmonary resuscitation (CPR) had been carried out immediately and continued until first medical contact (FMC), approximately 15 min after cardiac arrest. The initial assessment of the heart rhythm at FMC indicated ventricular fibrillation, which could be successfully terminated, and the heart rhythm stabilized by defibrillation and 300 mg IV amiodarone. After the return of spontaneous circulation (ROSC), the patient was transferred to the nearest district hospital. Unfortunately, a rapidly progressing cardiogenic shock developed over the next few hours. Due to bad weather conditions, immediate transfer to our University Cardiac Arrest Center had to be postponed. Therefore, the patient did not arrive at University Hospital Marburg until almost 22 h later, by now in ongoing cardiogenic shock for more than 12 h.

The patient’s family history reported no known co-morbidities, but the family reported that the patient had experienced episodes of syncope over the past 2 months. In this context, one-vessel coronary artery disease was diagnosed in November 2019, and a PTCA and a drug-eluting (DE) stent implantation in coronary ramus diagonalis 1 (RD1) were consequently carried out. No other structural heart dysfunction was diagnosed.

On arrival at University Hospital Marburg, the patient was in progressive, fulminant cardiogenic shock with a high-dose and further increasing catecholamine requirement (norepinephrine 200 μg/min, epinephrine 200 μg/min) to maintain a mean arterial pressure (MAD) of about 60mmHg. The clinical picture of a severely restricted respiratory situation emerged as pronounced pneumonia/ARDS (Horovitz Index: 74%, PaO_2_ 63%, FiO_2_ 85%). A whole-body CT scan revealed bilateral pulmonary infiltration in post-resuscitation, most likely due to aspiration and shock after cardio-pulmonary resuscitation. Other relevant reasons or consequences of cardio-pulmonary resuscitation could be excluded. The first blood-gas analysis showed a pronounced lactate acidosis (lactate>8 mmol/l). Procalcitonine was elevated by more than 100 μg/l, C-reactive protein (CRP) was 157 mg/dl, and leucocytes were 25 G/l. After the CT scan, the patient was immediately transferred to the cath lab for coronary angiography and implantation of a veno-arterial ECMO (vaECMO) in the event of hemodynamic and respiratory failure via the right *A. femoralis* (19F, 23 cm length), and the left V. femoralis (23F, 55 cm length) with antegrade perfusion of the *A. femoralis* via vaECMO side port. The implantation of the ECMO cannula was complicated by the very small and constricted vessels of this slim patient (62 kg, 173 cm), who was on high-dosage vasopressor therapy. The coronary angiography revealed only a minor residual in-stent stenosis of the rather small ramus diagonalis 1 (RD1), which did not require any further intervention. In transthoracic echocardiography, we saw a severely impaired systolic left-ventricular ejection function (LVEF) of about 30% in cardiogenic-septic shock post-resuscitation without any relevant valvular defects. After the patient’s transfer to the ICU, ballooning of the abdominal wall due to an abdominal compartment with intra-abdominal hypertension grade IV was diagnosed. The intra-abdominal pressure (IAP) was 39mmHg, most likely part of the intraperitoneal fluid accumulation and capillary leakage due to low blood flow and impaired organ perfusion in combined cardiogenic and septic shock.

In this situation, the performance level dramatically reduced, and the risk of failure of the venous backflow of vaECMO due to the enormous intraabdominal pressure increased drastically. Mean arterial blood pressure dropped immediately to 35–40mmHg. Despite the dramatic clinical situation, abdominal surgery to improve venous and arterial blood circulation was rejected because the mortality rate for grade IV abdominal compartment syndrome is >50%.^3^ It was decided to temporarily operate the vaECMO by the manual crank to maintain the function of the vaECMO, achieve sufficient arterial blood pressure, avoid blood clotting, and gain time for decision-making regarding the further therapy strategy for this young patient. The manual operation was briefly successful, but we needed to find a solution for establishing a consistent and sufficient vaECMO flow. Ultimately, we placed the inflow of the vaECMO drainage cannula maximally thoracic to the right atrium to avoid intra-abdominal pressure. This resulted in a longer-term improvement in performance level, with a still fluctuating but continuous vaECMO-blood flow of 2–2.5 L/min.

Thus, the overall hemodynamic situation was precarious for the following hours, despite the still administered high-dosage vasopressor and inotrope therapy (norepinephrine 164 μg/min, epinephrine 180 μg/min) and extracorporeal support of about 2 L/min. In addition, the respiratory situation (FiO2 90%, vaECMO O2: 100%) was still highly stressed.

**Figure 1.**
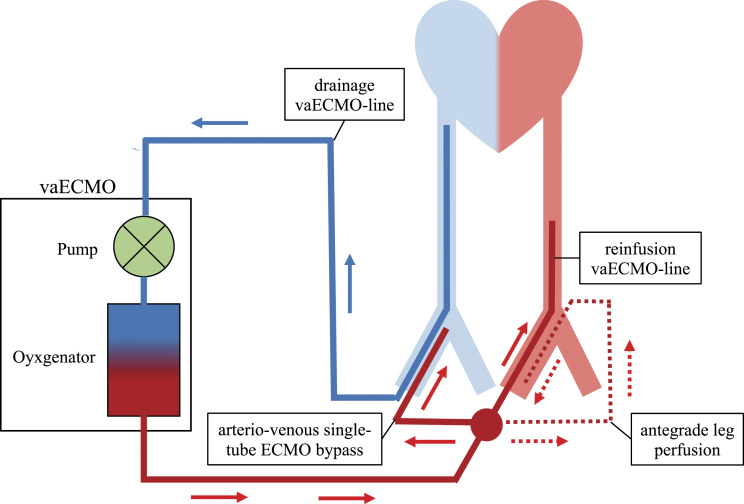
Schematic figure of the vaECMO system including the arterio-venous single-tube ECMO bypass. vaECMO: veno-arterial extracorporeal membrane oxygenation.

To further improve venous backflow and abdominal perfusion, especially the gut and liver perfusion, we decided to reverse the vaECMO perfusion without losing the cardiopulmonary support of the vaECMO. To that end, we placed a second 14F (30 cm length) venous line into the right femoral vein and connected this venous line with the 19F reinfusion vaECMO-line, thereby reversing abdominal perfusion and increasing venous backflow. This “arterio-venous shunt” immediately stabilized the patient by enhancing ECMO support from 2 L/min up to 3 L/min Figure 1. Fortunately, from then on, the vaECMO maintained a stable performance level (approx. 3–3.5 L/min), resulting in a constantly decreasing vasopressor support over the next 12 h (norepinephrine 30 μg/min, no epinephrine).

The respiratory situation also stabilized, and we gradually reduced the fraction of inspired oxygen (FiO2). Meanwhile, the intra-abdominal pressure decreased. Unfortunately, perhaps due to the prolonged need for high-dosage catecholamine therapy in cardiogenic shock, a probably vaECMO-associated disseminated coagulation disorder developed, causing micro-embolisms and vasospasm, and progressive ischemia of the lower extremities on both sides, despite antegrade leg perfusion via the right *A. femoralis*. After a significant reduction of catecholamine doses, the timely weaning off from the vaECMO was possible. Ultimately, the successful explantation of vaECMO was carried out only 7 days after the patient was admitted to Marburg hospital.

Unfortunately, the pronounced ischemia of the right lower leg and the left foot was irreversible. The lower extremities had to be amputated. The detection of multi-resistant *pseudomonas aeruginosa* in the surgical wound of the right leg led to multiple surgical revisions and antimicrobial therapy for several weeks.

In the context of multi-organ failure due to cardiogenic-septic shock and rhabdomyolysis (creatine-kinase 3476U/l, creatinine 3.5 mg/dl, urea 170 mg/dl, CRP 245 mg/L, leukocytes 14 G/l), renal replacement therapy was initiated. In the course of further treatment, kidney function was fortunately restored, and renal replacement therapy was not required. A secondary finding of the initial CT scan post-resuscitation was a fracture of the second lumbar vertebra, which was stabilized by our colleagues from neurosurgery.

The overall infective situation improved. However, respiratory weaning proved difficult due to ongoing severe pneumonia despite multiple antibiotic therapy regimes (ampicillin-sulbactam and levofloxacin, meropenem, and fosfomycin). In consequence, a tracheotomy had to be performed on day 15. After a total of 48 days of invasive ventilation, the tracheal cannula was removed. From then on, the patient could breathe on his own and independent of support. Fortunately, despite all the complications, the neurological outcome was completely adequate. The patient managed his extraordinary situation surprisingly well, considering the loss of both lower extremities. There was no evidence of post-traumatic stress disorder or depressive posture. The patient intended to return to his work as a computer scientist.

After sudden cardiac death a. e. due to myocarditis (LVEF 35% after several weeks on heart insufficiency therapy), a cardioverter defibrillator (ICD) was implanted for secondary rhythm prophylaxis. However, the persistent colonization of described multi-resistant *pseudomonas aeruginosa* and the limitations that have arisen due to the COVID-19 pandemic delayed the desired transfer to a rehabilitation clinic. Instead, intensive physical therapy was carried out at University Hospital Marburg. After a total of 111 days in ICU, we were able to discharge the patient to rehabilitation, with his condition restored as well as possible. At this time, the patient was already mobile in a wheelchair and able to cover a few meters independently. In addition, he had made significant progress with the gross and fine motor skills of his upper extremities through physio- and occupational therapy.

## Discussion

Despite the new therapeutic options regarding extracorporeal membrane oxygenation (ECMO), mortality from the consequences of cardiogenic and septic shock has remained high over the last few years.^4–6^ The goal is to initiate therapy before the onset of the “vicious circle” of cardiogenic shock resulting in multi-organ failure and death. However, device implantation itself does not guarantee successful therapy. The management of complications in the acute phase of a cardiogenic and septic shock continues to pose a significant challenge to modern device-assisted medicine.^7,8^

Abdominal compartment syndrome, that is, due to therapy-related intraabdominal fluid overload and capillary leakage, occurs in up to 10% of patients requiring vaECMO therapy.^9,10^ According to the current literature, decompressive laparotomy to prevent the threatening loss of function of the vaECMO is the proposed therapy in this time-critical situation.

In this case of a bail-out situation without any immediate surgical assistance available, we were able to show a less invasive method to prevent loss of ECMO function and multi-organ failure due to abdominal compartment by placing the drainage ECMO inflow cannula higher in the upper thorax and establishing an abdominal arterio-venous high-flow shunt via reperfusion ECMO cannula. Moreover, this alternative technique was able to restore vaECMO function quickly, without generating additional abdominal wounds because of abdominal catheter placement, which, in turn, might become a source of infection, prolonging septic conditions in this already life-threatening situation.

## Conclusion

Timely medical decisions and actions are obligatory in highly critical situations, such as an abdominal compartment under vaECMO therapy. The basic understanding of hemodynamics, the experience of the intensive care team, and a perhaps not entirely conventional, yet, in the end, very effective therapy saved the life of this young patient.
